# Prenatal and postnatal determinants in shaping offspring’s microbiome in the first 1000 days: study protocol and preliminary results at one month of life

**DOI:** 10.1186/s13052-020-0794-8

**Published:** 2020-04-15

**Authors:** Benedetta Raspini, Debora Porri, Rachele De Giuseppe, Marcello Chieppa, Marina Liso, Rosa Maria Cerbo, Elisa Civardi, Francesca Garofoli, Maria Cristina Monti, Mirco Vacca, Maria De Angelis, Hellas Cena

**Affiliations:** 1 Dietetics and Clinical Nutrition Laboratory - Department of Public Health, Experimental and Forensic Medicine, University of Pavia, via Bassi 21, 27100 Pavia, Italy; 2 National Institute of Gastroenterology “S. de Bellis”, Institute of Research, Castellana, 70013 Grotte, BA Italy; 3 European Biomedical Research Institute of Salerno EBRIS, 84125 Salerno, Italy; 4 Department of Soil, Plant and Food Science, University of Bari Aldo Moro, Bari, Italy; 5 Neonatal Unit and Neonatal Intensive Care Unit, Fondazione IRCCS Policlinico San Matteo, Pavia, Italy; 6 Department of Public Health, Experimental and Forensic Medicine - Unit of Biostatistics and Clinical Epidemiology, University of Pavia, 27100 Pavia, Italy; 7 Clinical Nutrition and Dietetics Service, Unit of Internal Medicine and Endocrinology, ICS Maugeri IRCCS, Pavia, Italy

**Keywords:** Gut microbiota, Pregnancy, Mode of delivery, Breastfeeding, Gestational exposures factors, Developmental origins of health and diseases (DOHaD)

## Abstract

**Background:**

Fetal programming during in utero life defines the set point of physiological and metabolic responses that lead into adulthood; events happening in “the first 1,000 days” (from conception to 2-years of age), play a role in the development of non-communicable diseases (NCDs). The infant gut microbiome is a highly dynamic organ, which is sensitive to maternal and environmental factors and is one of the elements driving intergenerational NCDs’ transmission.

The A.MA.MI (Alimentazione MAmma e bambino nei primi MIlle giorni) project aims at investigating the correlation between several factors, from conception to the first year of life, and infant gut microbiome composition. We described the study design of the A.MA.MI study and presented some preliminary results.

**Methods:**

A.MA.MI is a longitudinal, prospective, observational study conducted on a group of mother-infant pairs (*n* = 60) attending the Neonatal Unit, Fondazione IRCCS Policlinico San Matteo, Pavia (Italy). The study was planned to provide data collected at T0, T1, T2 and T3, respectively before discharge, 1,6 and 12 months after birth.

Maternal and infant anthropometric measurements were assessed at each time. Other variables evaluated were: pre-pregnancy/gestational weight status (T0), maternal dietary habits/physical activity (T1-T3); infant medical history, type of feeding, antibiotics/probiotics/supplements use, environment exposures (e.g cigarette smoking, pets, environmental temperature) (T1-T3). Infant stool samples were planned to be collected at each time and analyzed using metagenomics 16S ribosomal RNA gene sequence-based methods.

**Results:**

Birth mode (cesarean section vs. vaginal delivery) and maternal pre pregnancy BMI (BMI < 25 Kg/m^2^ vs. BMI ≥ 25 Kg/m^2^), significant differences were found at genera and species levels (T0). Concerning type of feeding (breastfed vs. formula-fed), gut microbiota composition differed significantly at genus and species level (T1).

**Conclusion:**

These preliminary and explorative results confirmed that pre-pregnancy, mode of delivery and infant factors likely impact infant microbiota composition at different levels.

**Trial registration:**

ClinicalTrials.gov identifier: NCT04122612.

## Background

Starting from the Barker Hypothesis, the recognition that the programming phenomenon concerned not only fetal life but also development, extending back into the early embryonic period and forward into infancy and early childhood, lead to the Developmental Origins of Health and Diseases (DOHaD) [1].

Evidence from DOHaD theory has pointed out biological mechanisms supporting the intergenerational nature of non-communicable diseases (NCDs), such as obesity, type 2 of diabetes, cancer, respiratory diseases, which are an increasing public health problem in most countries [2, 3]. Furthermore, scientific evidence confirms that the events occurring in the early stages of life play a critical role in fostering the development of chronic diseases throughout the life-course, indicating the high relevance of “the maternal environment” impact on the life of the future child [4, 5]. This critical period for offspring’s future health, known as “the first 1000 days”, begins at conception and continues until two years of life.

One of the key elements driving NCDs intergenerational nature and playing a pivotal role in “the first 1000 days”, is the gut microbiome, a highly dynamic organ which is sensitive to environmental factors and that modifies its composition over the host’s lifespan [6].

The bacterial establishment process is a complex phenomenon which begins in utero: in the past the intrauterine environment was perceived as sterile but recent evidence supports an intrauterine maternal-to-fetal exchange of microbes, challenging the traditional “sterile womb” that has been acknowledged worldwide for more than a century [7–9]. Most gut microbes are either harmless or have beneficial properties for the host and protect against invading pathogens; disruption of the normal balance within the gut microbiome (also called dysbiosis) has been associated with NCDs development [10].

Thus, understanding the underlying mechanisms that regulate the maintenance of a balanced microbiome may therefore have therapeutic implications or lead to strategies to decrease the risk of NCDs development [10].

Numerous factors are known to affect the diversity and architecture of the infant’s gut microbiome during the first year of life, such as maternal factors (e.g. Body Mass Index and weight fluctuation) before pregnancy [11], gestational exposures [12–15] (e.g. maternal lifestyle, weight gain, pathophysiology conditions, supplements use, antibiotics exposure, pollutants exposure), mode of delivery [16, 17] and type of feeding [18], triggering a gut microbiome dysbiosis [19].

Excessive weight gain during pregnancy is known to be one of the maternal factors affecting the gut microbial composition of the newborn to delivery to adulthood [11] and previous human studies showed that children born to women who were affected by obesity or overweight during pregnancy exhibited significant variations in gut microbiome composition at the different stages of life, when compared with those from normal-weight mothers [20, 21].

Another important and well-established factor influencing gut microbial composition of the newborn is the maternal dietary habits during pregnancy [22, 23]; it has been previously reported in in vivo models that gut microbiome dysbiosis in offspring is influenced by the western-style diet during pregnancy and lactation in different animal models [24, 25].

Several studies have identified the presence of different intestinal microbial profiles between vaginally delivered and cesarean section delivered babies [26], with a lower microbiome richness and diversity among infants born by elective cesarean section compared to those born vaginally [26].

Changes in the maternal microbiome composition could also be vertically transferred to the newborn, not only at birth but also during lactation, promoting the inoculum of an altered microbial community that may have short- and long-term health consequences [27]. Moreover, feeding mode (formula or breastfeeding) seems to have a great impact on the gut microbial composition, besides perinatal and other factors, including mode of delivery, gestational age and maternal weight status as well as life-style, would affect the microbial and non-microbial components of breastmilk, impacting infant gut and health [28]. Furthermore evidences show that breastfeeding is able to prevent the onset of dysbiosis and other pathologies (i.e. autoimmunity and allergic disorders), indeed the World Health Organization (WHO) recommends that infants should be exclusively breastfed for the first 6 months of life [29]. Furthermore, there are two major components to be considered for intestinal microbiome differences between different individuals: changes caused by age and effects induced directly by dietary changes [30].

**Fig. 1:**
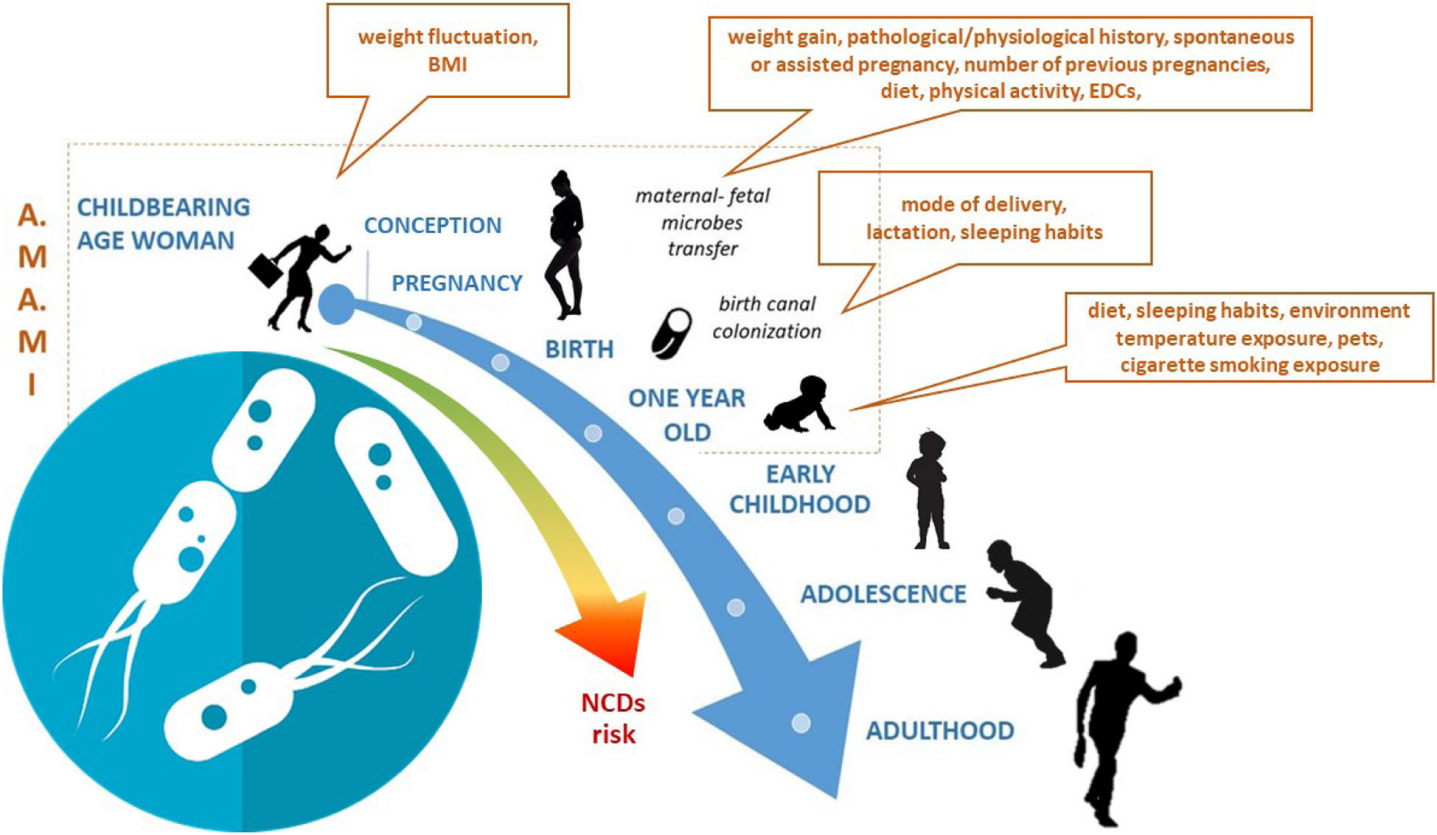
Conceptual framework of A.MA.MI study, adapted from Stiemsma et al. [8]

Based on all the aforementioned considerations, the A.MA.MI (*Alimentazione MAmma e bambino nei primi MIlle giorni*) project was designed with the purpose to investigate the possible correlation between maternal prenatal and gestational exposure factors, mode of delivery, lactation, family environment, infant diet, sleeping habits, and the infant gut microbiome composition, during the first year of life at different follow-up (Fig. 1).

In the present research article, the study design and the entire protocol of A.MA.MI are described. In addition, we present some preliminary and explorative results, concerning the investigation of the intestinal microbiome composition of infants at birth in the first month of life .

## Methods

### Study design and aims

A.MA.MI is a longitudinal prospective observational study that includes mother-infant pairs (dyads), from birth to 1 year of child’s age aimed at:

1. PRIMARY OUTCOME - investigating the intestinal microbiome development of infants (age: 0–11.99 months) by: i) identifying the relative abundance of the dominant microbes, ii) assessing the microbial diversity, and iii) assessing inter-individual variation.
2. SECONDARY OUTCOME – exploring the possible correlation between maternal factors before the pregnancy (weight status), gestational exposures (maternal diet, physical activity, weight status), mode of delivery, feeding mode (formula or breastfeeding), family environment exposure, infant diet, sleeping habits and infant intestinal microbiome composition, during the first year of life at different follow-up:

- immediately after the delivery (not more than 2 days after the delivery, T0);
- 1 month after birth (T1);
- 6 months after birth (T2);
- 12 months after birth (T3).

The study was approved by the Human Ethics Committee of Fondazione IRCCS Policlinico S. Matteo of Pavia (Protocol number: 20180022618; 6/12/2018) and it was conducted according to the Good Clinical Practice guidelines. The written informed consent of the parents/legal guardian was provided. The Human Ethics Committee of Fondazione IRCCS Policlinico S. Matteo of Pavia approved this procedure.

#### Participants

In the present study, the recruitment of 63 dyads started, with the purpose to complete the study with a minimum of 50 mother-infant pairs (mother/new-born), attending the Neonatal Unit, Fondazione IRCCS Policlinico San Matteo, Pavia (Italy) and according to the following inclusion criteria:

- Infants of both sexes born to natural or caesarean delivery;
- Gestational age between 37 and 42 completed weeks;
- Italian-speaking parents;
- Ability of the parent/guardian to give informed consent;
- Ability of the mother to respond to the structured interview/questionnaires;

Exclusion criteria:

- Infants or mothers with genetic/congenital diseases;
- Infants hospitalized in neonatal intensive care unit immediately after birth;
- Infants selected for other clinical studies;
- Presence of gestational diabetes;
- Presence of hyperthyroidism during pregnancy;

Before the delivery, during the routine visit of the mother, the parent /legal guardian were informed about the nature of the study and the possibility of their active involvement in and, then, they were invited to sign the informed consent.

Antibiotic intrapartum prophylaxis was conducted according to the American College of Obstetricians and Gynecologists (ACOG) guidelines 2019 [31].

#### Questionnaires and interviews

**Fig. 2:**
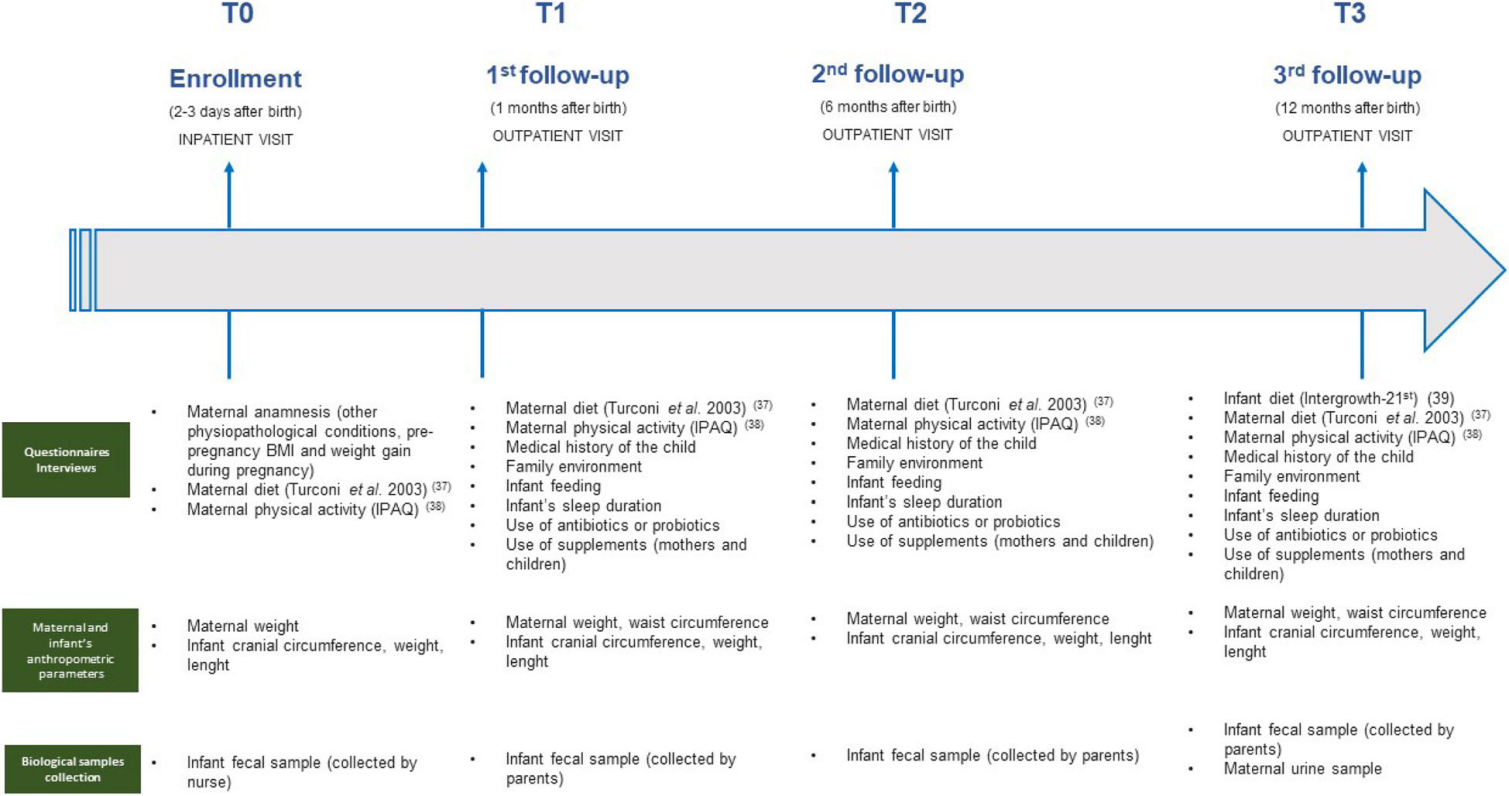
Timeline and data collection

The timeline of all data collection is shown in Fig. 2.

At T0 mothers are interviewed by trained personnel of the research group about:

- their pathological/physiological history, information about pregnancy (type of current pregnancy, spontaneous or assisted pregnancy, number of previous pregnancies) and anthropometric parameters (height, weight pre and post-pregnancy, Body Mass Index (BMI) pre and post- pregnancy);
- their dietary habits by means of a previously validated questionnaire [32];
- their physical activity habits by means of a previously validated questionnaire [33].

Mothers are also interviewed at T1, T2 and T3 about:

- medical history of the child, family environment (how many people live in the house, presence of pets, temperature, socio-economic level of the family, infant’s exposure to passive smoking), feeding mode (formula or breastfeeding), time and characteristics of weaning, infant’s sleep duration, use of antibiotics or probiotics and use of supplements both in mothers and children;
- their dietary habits by means of a previously validated questionnaire [32].
- their physical activity habits by means of a previously validated questionnaire [33].

Mothers are also interviewed at T3 in order to investigate infant’s dietary habits at 1 year of age, by using a previously validated questionnaire [34], adapted using the forward-back translation method (https://www.who.int/substance_abuse/research_tools/translation/en/).

Particularly, in order to investigate maternal diet (T0-T3), two sections of an adapted version of a previously validated questionnaire [32] have been identified and administered to the mothers.

The Food Frequency section (FF) assesses the daily consumption of typical food and beverages such as pasta, cereal products, bread, vegetables and fruits, milk and yogurt, and weekly consumption of meat and meat derivatives, legumes, fish, eggs and cheese; alcoholic beverage, sweets and desserts consumption were also investigated [32]. The Dietary Habit section (DH), investigates food habits related to meals frequency, number of portions, water consumption as well as soft drinks and alcoholic beverages [32]. Each section consists of questions with the following response categories: always, often, sometimes, never. The score assigned to each response ranges from 0 to 3, with the minimum score assigned to the less healthy choice and the maximum score assigned to the healthiest one according to the National Dietary Guidelines [35].

To investigate physical activity, the short version of a validated questionnaire on physical activity and sedentary behavior assessment (International Physical Activity Questionnaire, IPAQ) [33] is adopted. IPAQ is an instrument developed and tested for the adult population (age range of 15–69 years) surveillance of physical activity. The IPAQ short version (7 items) is structured to provide information and separate scores about the time spent in vigorous, moderate intensity activity, in walking, and in sedentary activity. Estimate of the total score for the short form requires the sum of the frequencies (in days) and duration (in minutes) of those activities; each activity is expressed by its energy requirements defined by METs to produce a score in MET-minutes.

The selected MET values are obtained using the Ainsworth et al. Compendium [36] and an average MET score is achieved for each type of activity. For example, all sorts of walking are included, and a medium MET value for walking is created. The same method is used for moderate-intensity and vigorous-intensity activities. Note that questions about resting time are developed as separate indicators and not as part of the summed physical activity score.

Finally, to investigate infant’s dietary habits at 1 year of age (T3) both two sections of an adapted version of a previously validated questionnaire are used [34]. Section 1 investigates infant feeding during the first year of life starting from the discharge from the hospital, while section 2 is composed by a Food Frequency questionnaire for food consumption assessment over the past 28 days [34].

#### Maternal and infant’s anthropometric parameters

Maternal anthropometric parameters, such as height (cm) and weight (kg) were planned to be measured at T0-T3 and BMI (Kg/m^2^) was then calculated; waist circumference (WC) (cm) was planned to be measured at T1-T3 (Fig. 1).

Weight and height were measured with standardized procedures. Body weight was measured with mothers wearing only their underwear and without shoes by means of a steelyard scale (precision ±100 g); body height was measured on mothers without shoes by means of a stadiometer (precision ±1 mm). WC was measured to the nearest centimeter with a flexible steel tape measure with participants standing, with crossed arms, placing the hands on opposite shoulders. After gently exhaling, the abdominal WC was measured on the horizontal plane between the lowest portion of the rib cage and the uppermost lateral border of the right ilium [37].

BMI was also calculated as a ratio between weight and height squared with weight in kilograms and height in meters [37].

Each child was planned to be visited before discharge from the hospital (T0), at 1 month (T1), at 6 months (T2) and at 1 years of age (T3) (Fig. 1). Infant weight (kg), length (cm) and head circumference (cm) were also measured (T0-T3) with standardized procedures [37].

Body weight was measured with naked infants by means of a steelyard scale (precision ±100 g). Length was measured laying infants on an infantometer with the feet toward the mobile foot piece and the head against the fixed headpiece by the examiner, who positioned the feet and ensured that the head was lying in the Frankfort horizontal plane. If it was impossible to reach both legs outstretched in the correct position, the examiner ensured that at least one leg was straight with the foot flexed against the foot piece.

The infants head circumference was measure to the nearest 1 mm using non-stretch measuring tape which placed around the infant’s head with the tape adherent to the frontal bones of the skull; slightly above the eyebrows; perpendicular to the long axis of the face; above the ears; and over the occipital prominence at the back of the head, moving the tape up and down over the back of the head to locate the maximum circumference and tightening the insertion tape to fit it perfectly around the head and compress the hair and underlying soft tissue, according to standard assessment method [37].

#### Samples collection

For each infant stool samples were collected (T0 - T3) into 2 different screw-top containers and labeled with the newborn’s unique study ID number. One sample was stored at − 80 °C at Neonatal Intensive Care Unit, IRCCS Policlinico San Matteo Foundation (Pavia, Italy); the other one was stored at − 80 °C at the Institute of Human Anatomy, Department of Public Health Experimental and Forensic Medicine, School of Medicine, University of Pavia (Pavia, Italy), until analyzed.

Particularly, at T0, a trained nurse collected a stool sample of the newborn. At the end of the visit, a member of the research team scheduled the second well-child-visit (1 month from the birth, T1) and gave to the parents/legal guardian a kit for the collection of the second stool sample of the newborn. The parent/legal guardian was also instructed how to collect stool samples independently at home.

The infants’ parents were contacted by a research team member 1–2 days before the scheduled visit at T1 to check the health of the new-born; besides, they were asked to submit a stool sample at another time if:

- child was currently ill with fever, respiratory illness, or gastrointestinal illness;
- child had a diarrheal illness lasting more than 24 h in the past 7 days;
- child has been treated with antibiotics in the past 7 days.

The stool sample collection at the other two follow-up visits (T2, and T3) followed the same procedures.

### Samples analysis

Stool samples were shipped on dry ice to Genomix 4 Life Srl (C/O Laboratory of Molecular and Genomic Medicine - Campus of Medicine and Surgery, Baronissi, Salerno, Italy, spin-off of the University of Salerno, Fisciano, Italy) where 16S metagenetic analysis was carried out. Samples had only the study ID number; no clinical or personal information was shipped with the samples.

#### DNA extraction from stool

Total genomic bacterial DNA was isolated from frozen stool samples using the QIAamp® Fast DNA Stool Mini Kit (QIAGEN, Hilden, Germany), according to the manufacturer’s instructions.

#### Gut microbiota estimated by 16S rRNAs metagenetic analysis

16S metagenetic analysis was conducted using the Illumina MiSeq platform. The V3-V4 region of the 16S rRNA gene was amplified for analysis of diversity inside the domains of Bacteria [38]. PCR and sequencing analyses were conducted according to the protocol of Genomix4life. Quality control (QC) and taxonomic assignments were done according to the QIIME and the Ribosomal Database Project Bayesian classifier in combination with a set of custom designed informatics pipelines implemented by Genomix4life for analyses of microbial communities.

Taxonomic attribution was carried out using the BLAST search in the NCBI 16S ribosomal RNA sequences database [39]. The percentage of each bacterial operational taxonomic units (OTUs) was investigated separately for each sample, providing relative abundance data among the samples based on the relative numbers of reads within each [39]. Alpha diversity (observed species, Chao1 richness and Shannon diversity indices) was calculated using Qiime Variations [40, 41] in microbial communities between groups; communities were studied using the phylogeny-based, un-weighted Unifrac distance metric [41].

### Statistical analysis

#### Power analysis

The A.MA.MI study was initially conceived on a relatively small scale (at least 60 dyads), in order to complete the study with a minimum of 50 mother-infant pairs, and provide the initial feasibility of the A.MA.MI study protocol (it may be considered a pilot study). The aim is to explore the microbiome variability in infants and verify the magnitude of effects of several maternal and infants’ factors possibly associated to microbiome development. The information obtained will be also used to better define the sample size of a bigger study to validate the results of the former study. Therefore, we will calculate and mention *post-hoc* power, when discussing specific results at the end of the pilot study.

To minimize losses during follow-up, researchers planned to keep participants informed and to remind all the follow-up’s activities throughout phone-calls.

#### Primary and secondary objective analysis plan

Data are summarized by means of descriptive statistics, such as means and standard deviations or median and interquartile range (IQR), as appropriate, for quantitative variables and relative frequencies for qualitative ones.

To investigate the intestinal microbiome development of infants, principal component analysis (PCA) and Spearman correlation has been used to assess changes in the microbial composition across different follow-up times.

To evaluate the association of the intestinal microbiome composition with the maternal and infants’ factors previously described, t-tests/ANOVA or Mann-Whitney/Kruskal-Wallis have been chosen, as appropriate. The relative abundances of different phyla and genera and diversity indexes have been compared across groups defined by presence/absence or different categories of the prenatal or postpartum specific factor.

In order to capture the characteristic of excess zeros at lower taxonomic levels and model the screwed abundance of bacteria data, a zero-inflated model has been also implemented and results compared.

To evaluate the association of the intestinal microbiome development and the condition of adverse growth parameters, repeated-measure ANOVA or Friedman tests have been used, with adjustment for multiple comparisons. A post-hoc multilevel mixed-effects linear regression test has been performed in case of group-time interaction.

Ultimately, to define the relationship between intestinal microbiome composition and development and prenatal or neonatal factors, generalized linear models (GLM) using appropriate link functions to relate the model to the different dependent variables, have been performed. All regression models could be carried out adjusting for possible confounding factors, as for example socio-demographic information. All the tests are two tailed, and statistically significant level set at 0.05.

All analyses have been performed using STATA 15.

## Results

The enrolment started in October 2018 then data and samples at T0, T1 and T2 were collected. All the analysis regarding T2 and the enrolment at T3 are on-going.

Below some preliminary and explorative results, concerning the investigation of the intestinal microbiome composition of infants at T0 and at T1, related to the maternal weight status, type of delivery and lactation, are presented.

### Dyads general characteristics

A total of 63 dyads, attending the Neonatal Unit, IRCCS Policlinico San Matteo, Pavia (Italy) were recruited, according to one dyad dropped-out after the enrolment while four dyads dropped-out at T1.

The description of the dyads’ general characteristics is presented in Table 1.

**Table 1:** General characteristics of the dyads. Data are presented as median and InterQuartile Range (IQR)

Dyad	Parameters	median	IQR
**Mothers** (*n* = 63)	**Age** (years)	33.0	29.0–37.0
	**Pre- pregnancy weight** (kg)	60.0	53.0–65.5
	**Pre -pregnancy height (cm)**	164.0	160.0–170.0
	**Pre-pregnancy BMI** (Kg/m^2^) • 65.6% (*n* = 42): normal weight, • 26.3% (*n* = 16): overweight/obesity • 8,2% (n = 5): underweight.	22.0	19.5–25.0
	**Weight gain during pregnancy** (Kg) • 54.1% (*n* = 33): adequate weight gain during pregnancy ^a^	12.5	10.0–15.0
**Infants** (n = 63; 31 M/32 F)	**Gestational age at birth** (weeks)	40.0	39.0–41.0
	**Birth weight** (Kg)	3.3	3.1–3.5
	**Length** (cm)	50.0	50.0–52.0
	**Cranial circumference** (cm)	34.0	33.5–35.0

^a^ according to the Institute of Medicine (US), guidelines [Institute of Medicine, US; Weight gain during pregnancy: re-examining the guidelines. Washington, DC. National Academies Press; National Academy of Sciences]

Thirty-six women (48.1%) enrolled were at their first pregnancy. The type of conception was spontaneous in most of women 96.8%, (*n* = 60) and only 3.2% (*n* = 2) underwent an assisted reproductive technologies (ART). The delivery mode was spontaneous vaginal delivery in 85.5% of women (*n* = 53).

The 58.6% of infants (*n* = 34) were breast-fed while the 41.1% (*n* = 24) were formula-fed.

### Infant microbiome analysis

Preliminary results are reported describing the gut microbiome composition of the new-borns at T0 (meconium) and at T1.

Of 118 neonatal stool samples, the average of reads which passed filter quality was 106,298.81 ± 69,276.42 sequences. The bacterial community was analyzed considering alpha diversity (number of identified species OTUs) and Shannon diversity index (Additional file 1: Table S1).

#### Comparison of newborns vs 1 month old infants

The average number of OTUs found at T0 was greater than T1 (*p* = 0.001), while the species diversity (Shannon index) was comparable (*p* > 0.05) considering T0 versus T1.

Firmicutes, Bacteroidetes, Proteobacteria, Actinobacteria, Fusobacteria, Cyanobacteria and Verrucomicrobia represented more than 98% of 16S rRNA gene sequences in all samples. Sequenced phyla with relative abundance < 0.1% were grouped together in a category called *others*.

**Fig. 3:**
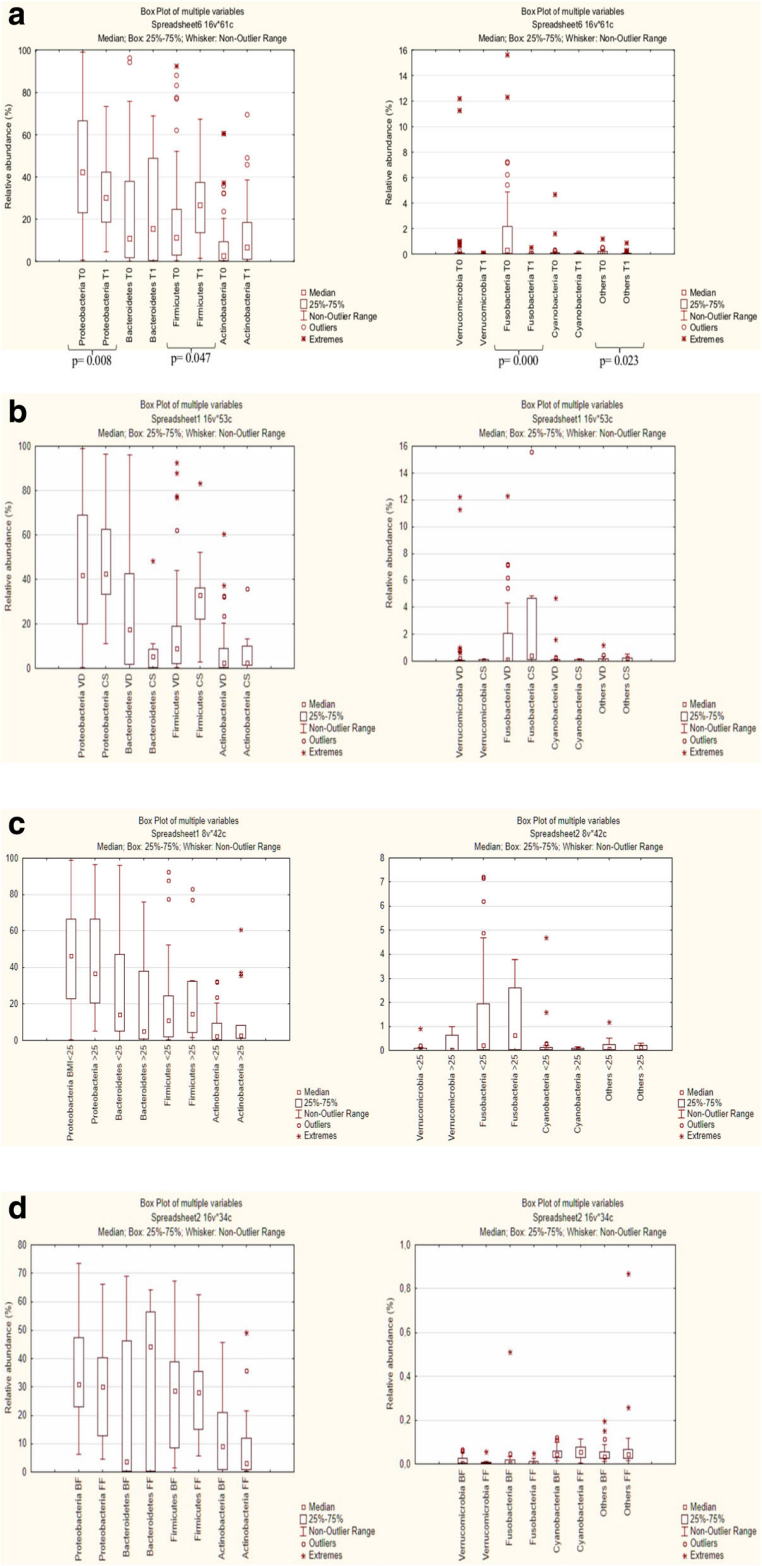
Relative abundance (%) of total bacteria (16S rRNA) found at phylum level in fecal samples of neonates. Phyla with relative abundance < 0.1% in all samples were grouped together in “Others”. Box plots showed: **a**) total bacteria found at T0 (meconium) and T1 (1 month of age); **b**) total bacteria found in the meconium of neonates born via cesarean section (CS) or vaginal delivery (VD); **c**) total bacteria found in the meconium of neonates born to normal weight (BMI < 25 Kg/m^2^) or mothers with overweight or obesity (BMI ≥25 Kg/m^2^) and **d**) total bacteria found in T1 samples of neonates fed with formula (FF) or breastfeeding (BF). (*p = *p*-value)

The relative abundance of Firmicutes increased at T1 if compared to meconium (T0: 19.71%, T1: 27.52%; *p* = 0.047), related to a decrease of Proteobacteria (T0: 46.23% vs. T1: 33.56%; *p* = 0.008) and Fusobacteria (T0: 1.65% vs. T1: 0.02; *p* = 0.000) (Fig. 3a).

At T1, at genus and species levels, a clear decrease of genera and species of Fusobacteria (i.e. *Fusobacterium naviforme, F. nucleatum*, *Leptotrichia shahii*, *L. wadei* and *Sebaldella*; *p* < 0.001) was found. On this way, in the cluster 1 of the permutation analysis performed with all stool samples (Figure S1a) was characterized by a positive score of amounts of the aforementioned genera.

Among Firmicutes, *Blautia*, *Lactobacillus* species (*L. gasseri* and *L. taiwanensis*), *Ruminococcus* (*R. gnavus*) and *Veillonella* (i.e. *V. atypica, V. criceti,* and *V. dispar*) showed a marked increase at T1 (*p* < 0.035).

Otherwise, *Streptococcus* spp. showed different trends among the species; indeed, some species decreased (i.e. *S. infantis*, *S. intermedius*, *S. sanguinis* and *S. tigurinus*) (*p* < 0.009), while other species increased 30 days after birth (T1), including *S. fryi* and *S. vestibularis* (*p* < 0.046).

Similarly, some genera of the Actinobacteria, i.e. *Actinomyces*, *Corynebacterium*, *Mycobacterium*, *Parascardovia*, *Propionibacterium*, and *Rothia* decreased comparing T1 against meconium (*p* < 0.041). On the other hand, *Collinsella* (T0: 0.05%, T1: 0.56%; *p* = 0.047) and especially *Bifidobacterium*, both genera of Actinobacteria, showed a marked increase (T0: 1.93%, T1: 12.39%; *p* = 0.000) after 1 month of age, as showed in the cluster 2 of permutation analysis performed with all stool samples (Figure S1a). While, comparing the cluster 1 (Figure S1a), which mainly included the T0-samples, to the cluster 2, which included the 98.25% of the T1-samples (56/57), it was observed that several species of Proteobacteria showed a significant decrease after birth, including Burkholderiaceae (*Lautropia spp.* and *Ralstonia spp.*) and Neisseriaceae (*Kingella spp.* and *Neisseria spp.*), as well as *Bradyrhizobium* spp., *Campylobacter* spp., *Methylobacterium* spp., *Oxalobacter* (especially *O. vibrioformis*), and *Pseudomonas* spp. (especially *P. brenneri*) (*p* < 0.037). On the contrary, only *Trabulsiella* (Enterobacteriaceae) and *Sutterella* (Sutterellaceae), both genera of Proteobacteria, were higher after 30 days comparing to meconium (*p* < 0.048). No significant increase was found among genera of Bacteroidetes, whereas *Capnocytophaga*, *Chryseobacterium*, *Porphyromonas*, *Prevotella*, and *Tannerella* were lower at T1 than T0 (*p* < 0.011).

#### Comparison of delivery mode at T0: vaginal delivery vs cesarean section

At phylum level no statistical differences were found between the meconium of neonates born via cesarean section (CS) vs. vaginal delivery (VD) (Fig. 3b). The main differences between the two sampled groups were found at genus and species levels.

Several genera and species of Firmicutes were higher in the meconium of neonates born via CS than VD, including Lactobacillaceae, particularly *Lactobacillus* and *Pediococcus, Bacillus* (especially *B. butanolivorans*), *Selenomonas* (*S. artemidis, S. infelix,* and *S. noxia*) (*p* < 0.025), and *Staphylococcus* (VD: 0.43%, CS: 9.41%; *p* = 0.000). On this way, the 77.78% (7/9) of the meconium-samples of neonates born via CS were clustered differently than VD-newborns, which were all grouped together in the cluster 1, as consequence of the high amounts of the aforementioned genera (Figure S1b). Furthermore, species of *Streptococcus* (*S. anginosus, S. intermedius, S. oralis*, and *S. tigurinus*), *Veillonella* (*V. atypica* and *V. denticariosi*) and *Clostridium* (i.e. *C. histolyticum*, *C. neonatale,* and *C. paraputrificum*) were detected higher in the CS-group (*p* < 0.035).

Among Actinobacteria and Proteobacteria, the OTUs that showed statistical differences (*p* < 0.05) in relationship with the delivery-mode were all higher in the CS group, in particular *Corynebacterium spp.* (*p* < 0.048), *Enterobacter spp.* (*p* < 0.02)*, Erwinia billingiae* (*p* = 0.045)*,* and *Lautropia mirabilis* (*p* = 0.038)*.*

#### Comparison of adequate maternal pre-pregnancy BMI vs high maternal pre-pregnancy BMI at T0

No statistical differences were found at phylum level (Fig. 3c), comparing the meconium of neonates born to pre-pregnancy normal-weight mothers (BMI < 25 Kg/m^2^) vs. mothers with overweight or obesity before pregnancy (BMI ≥25 Kg/m^2^).

Differently, at genus level, neonates born to mothers with BMI ≥ 25 Kg/m^2^ presented higher abundances of *Streptococcus* (*p* = 0.026), *Propionibacterium* and *Actinomyces* (Actinobacteria; *p* < 0.04), and *Kingella* and *Hylemonella* (Proteobacteria; *p* < 0.045). At species level, *Alkaliphilus peptidifermentans* was higher in neonates born to mother with overweight/obesity than those born to mothers with normal weight (0.78 and 0.13%, respectively; *p* = 0.016); similarly, within *Klebsiella*, 3 species (*K. variicola*, *K. granulomatis*, and *K. pneumoniae*) were significantly more abundant in meconium of neonates born to mother with overweight/obesity than those born to mothers with normal weight (*p* < 0.023).

#### Comparison of breastfeeding vs formula feeding at T1

No statistical differences were found at phylum level also among the T1-fecal samples of infants fed with formula (FF) compared to breastfed (BF) (Fig. 3d).

The permutation analysis (Figure S1d) showed, among the statistically different genera (*p* < 0.05), that the intestinal microbiota of neonates fed with formula differed from BF-group by a greater relative abundance of *Butyrivibrio* and *Faecalibacterium* (Firmicutes; *p* < 0.034), *Kitasatospora* (Actinobacteria; *p* = 0.044), *Paraprevotella* (Bacteroidetes; *p* = 0.046), and *Trabulsiella* (Proteobacteria; *p* = 0.02). Contrarily, the cluster 2, which included the 94.12% (32/34) of the stool samples of BF-infants, showed a negative normalized score of the relative abundance of the aforementioned genera.

At species level two species of *Bacteroides* (*B. rodentium* and *B. uniformis*) and two of *Parabacteroides* (*P. goldsteinii* and *P. merdae*) were found higher in FF than BF (*p* < 0.042). Similarly, among Firmicutes, *Butyrivibrio proteoclasticus*, *Enterococcus casseliflavus* and *E. gallinarum*, *Veillonella atypica* and *V. montpellierensis* were higher in FF (*p* < 0.05). Among Proteobacteria, only *Citrobacter werkmanii* and *Enterobacter aerogenes* were different between the two-sampled group (p < 0.05). No statistical differences were found among species of Actinobacteria and among minor phyla.

## Discussion

In the present research some preliminary results on the intestinal microbiome composition of infants at birth and at one month of age, considering factors such as, maternal pre-pregnancy BMI (BMI < 25 Kg/m2 vs BMI ≥25 Kg/m^2^), type of delivery (VD vs CS) and lactation (breastfed vs formula fed), are discussed.

Our results showed that at T0, the meconium was characterized by a greater predominance of Proteobacteria, especially species belonging to the genus *Escherichia*. Previous studies showed that the facultative anaerobes thrive in the primitive gut, thereby pointing toward a relatively aerobic intestinal environment [19, 42]. In this way, facultative anaerobes (such as members of the Enterobacteriaceae) find the optimal conditions for growth [43], whereas Firmicutes, Bacteroidetes, and Actinobacteria take over the predominance later on during infancy [44, 45]. The same evolution of intestinal microbial environment was observed also in our study, where the relative abundance of Firmicutes significantly increased at T1, if compared to meconium, while Proteobacteria and Fusobacteria significantly decreased.

Early-life microbes colonization is strongly influenced by birth mode that could influence the infant’s microbiome composition some years after birth [46].

The microbiome composition of a baby born by VD is predominantly influenced by the gut and vaginal maternal microbiome while the microbiome composition of a newborn delivered by CS is mainly influenced by the microbes of the surrounding environments species (e.g. hospital environment) and by the mother’s skin microbes [17]. Several studies have identified the presence of a different intestinal microbial profile between vaginally and CS delivered babies, with a gut microbiome richness and diversity lower in infants born by elective CS compared to those born vaginally [17]. In our sample, neonates born to CS showed a greater relative abundance of facultative aerobes such as *Clostridium*, *Corynebacterium*, *Staphylococcus*, and *Streptococcus *probably derived from maternal skin, hospital environment or hospital staff. Moreover, some studies suggested that aberrant gut microbiome composition, observed in early life of infants born via CS, may explain the higher risk to developing obesity later in life [19, 42, 47]. Particularly, it has been proposed that children born via CS might have a gut microbiome with a higher capacity to harvest food energy, thereby predisposing them to develop overweight or obesity [48].

The microbial composition of meconium of neonates born to mothers with pre-pregnancy BMI < 25 Kg/m2 as well as pre pregnancy BMI ≥ 25 Kg/m2 was also investigated. Maternal overweight, before and during pregnancy, is related to adverse outcomes for mothers and newborns; obesity during pregnancy is strongly associated with increased maternal and neonatal morbidity including gestational diabetes, preterm birth, infants born large-for-gestational-age (LGA), preeclampsia, and congenital anomalies [49]. Previous human studies showed that children born to women who were affected by overweight or obesity during pregnancy, exhibited significant variations in gut microbiome composition at different stage of life [14, 50, 51]. Collado et al. [14] reported that mothers with overweight had higher levels of *Bacteroides* genus in the third trimester of pregnancy. The higher levels of *Bacteroides*, among women with overweight in pregnancy, may be vertically transmitted to the newborn during VD. In fact, previous studies reported that, among VD neonates, the relative abundance of gram-negative *Bacteroides* spp. resulted more associated to infants born to mothers affected by overweight or obesity, than to those born to normal weight mothers [52]. Contrarily, it was shown that maternal pre-pregnancy BMI was negatively associated with fecal levels of *Bacteroides* when the infants were 1 month of age [14]. In contrast with previous findings [14, 50, 51], in the present study differences in the relative abundance of *Bacteroides* spp. were not detected. More studies are needed to clarify the specific changes in offspring microbiome composition related to maternal pre-pregnancy BMI and mode of delivery.

The main differences between the two groups (pre-pregnancy BMI < 25 Kg/m2 and pre pregnancy BMI ≥ 25 Kg/m^2^) were related to the relative abundance of specific genera and species. Interestingly, ¾ of the statistically different species belonged to *Klebsiella* genus and were higher in samples of neonates born to mothers with a pre-pregnancy BMI ≥ 25 Kg/m^2^. Actually, *Klebsiella* remains among the principal causes of nosocomial infections, since frequently develops multidrug resistance [53].

Higher Firmicutes levels and a higher ratio Firmicutes/Bacteroidetes were often observed in subjects affected by overweight or obesity [54]. Among Firmicutes, we found an over representation of *Streptococcus* in infants born to mothers whit a pre-pregnancy BMI ≥ 25 Kg/m^2^, in contrast with the recent study of Kameron Y. Sugino et al. [55].

Undoubtedly, an evaluation of fecal microbiota along time might explain how maternal pre-pregnancy BMI is transmitted to the infants and how this will affect their health.

Feeding type is another major factor triggering early microbial shaping and differences in the gut microbial composition between BF and FF infants are well documented [54]. Results from previous studies showed high levels of Bifidobacteria in BF infants, who also exhibit lower diversity than FF infants [56, 57]. Furthermore, the microbiome composition in early infancy is mostly dominated by species involved in human milk oligosaccharide (HMO) metabolism in breastfed infants [58]. These bioactive compounds seem to be beneficial to newborns as they not only promote healthier growth but also strengthen the immune system, they provide protection against allergies and may also offer protection from NCDs (such as type-2-diabetes, obesity), coeliac disease, diarrhea and many other metabolic disorders [59–63]. This is one of the main reasons why the World Health Organization (WHO) recommends that infants should be exclusively breastfed for the first 6 months of life [30].

It has been previously estimated that 25–30% of infant bacterial microbiome originates from breast milk, while around the 10% from bacteria from the skin of the breast [64]. On the contrary, it was previously demonstrated that the intestinal microbiome of infant’s formula-fed were characterized to greater relative abundance of *Enterococcus*, *Bacteroides*, and *Veionella * [28, 65]. In contrast with previous studies, we did not find substantial differences in terms of intestinal microbial composition at phylum levels, with some exceptions at genus and species levels comparing BF infants to FF infants (Fig. 3d).

### Strength and limitation

In general, one of the most outstanding strength of A.MA.MI is the great heterogeneity of information covering a lot of aspects of maternal and infant lifestyle end environment exposure factors, obtained about participants during the first year of life. The data collection and interpretation throughout questionnaires and structured interviews, as well as the biological samples collection and their analysis and results’ interpretation, need the presence of a multidisciplinary team with different expertise in the field of pediatrics, clinical nutrition, microbiology and biostatistics. Furthermore, the assessment of some pre-pregnancy factor, such as BMI, confirms the influence of maternal weight and lifestyle on the shaping of microbiota in the newborn.

Nevertheless, some limitations should be considered. The small sample size of study population and the short period that covers only one year after birth. Enlargement of the cohort and a longer follow-up, ideally to adulthood, will be considered. Finally, only the infant microbiome, and not the maternal one, was planned to be evaluated.

## Conclusion

These preliminary and explorative results confirmed that pre-pregnancy, maternal and infant factors likely impact infant microbiota composition at different levels. In particular, we found that the relative abundance of Firmicutes increased after one month of life (T1), if compared to meconium (T0), related to a decrease of Proteobacteria and Fusobacteria. Furtheremore, significant differences at genus and species levels were found concerning birth mode (cesarean section vs. vaginal delivery), maternal pre pregnancy BMI (BMI < 25 Kg/m^2^ vs. BMI ≥ 25 Kg/m^2^) and according to type of feeding mode (breastfeeding vs. formula-feding).

This study is an ongoing longitudinal prospective and observational study, and although it doesn’t cover the “1000 day of life” it contributes to the current understanding of how early pre-pregnancy, pre-natal and peri-natal factors may affect the intestinal microbiome composition and, consequently, future host health. Besides, the knowledge obtained will be used to plan a future prospective observational cohort, to be carried out over a longer period of time, aiming to identify risk factors for particular conditions and design tailored intervention programs for women of childbearing age planning a pregnancy.

## Supplementary information

**Additional file 1: Table S1**. Means of number of Reads Passing Filter quality, percentage of Reads classified to Genus level, Shannon diversity index, and identified species (OTUs) found: A) in the meconium (T0) and in fecal samples after 1 month (T1) of neonates; B) in the meconium of neonates born via cesarean section (CS) or vaginal delivery (VD); C) in the meconium of neonates born to normal weight (BMI < 25 Kg/m^2^) mothers affected by overweight or obesity (BMI ≥25 Kg/m^2^)), and D) in T1-samples of neonates fed with formula (FF) or breastfeeding (BF).

**Additional file 2: Figure S1a.** Permutation analysis summarizing the genera with relative abundance > 0.1%, statistically different (*p*-value< 0.05; Student’s t-test), found in the meconium of neonates (T0) and after 1 month of age (T1). **Figure S1b.** Permutation analysis summarizing the genera with relative abundance > 0.1%, statistically different (p-value< 0.05), found in the meconium of neonates born to vaginal delivery (VD) or via cesarean section (CS; highlighted in the red boxes). **Figure S1c.** Permutation analysis summarizing the genera with relative abundance > 0.1%, statistically different (*p*-value< 0.05), found in the meconium of neonates born to normal weight (BMI < 25 Kg/m^2^) or mothers affected by overweight or obesity (BMI ≥25 Kg/m^2^; highlighted in the red boxes). **Figure S1d.** Permutation analysis summarizing the genera with relative abundance > 0.1%, statistically different (*p*-value< 0.05), found in fecal samples of neonates after 1 month (T1) fed with breast milk (BF) or formula (FF; highlighted in the red boxes).

**Additional file 3: Figure S2a**. Permutation analysis summarizing the species with relative abundance> 0.1%, statistically different (*p*-value< 0.05; Student’s t-test), found in the meconium of neonates (T0) and after 1 month of age (T1). **Figure S2b**. Permutation analysis summarizing the species with relative abundance> 0.1%, statistically different (p-value< 0.05; Student’s t-test), found in the meconium of neonates born to vaginal delivery (VD) or via cesarean section (CS; highlighted in the red boxes). **Figure S2c.** Permutation analysis summarizing the species with relative abundance> 0.1%, statistically different (p-value< 0.05; Student’s t-test), found in the meconium of neonates born to normal weight (BMI < 25) or overweight/obese mothers (BMI ≥25; highlighted in the red boxes). **Figure S2d**. Permutation analysis summarizing the species with relative abundance> 0.1%, statistically different (p-value< 0.05; Student’s t-test), found in fecal samples of neonates after 1 month (T1) fed with breast milk (BF) or formula (FF; highlighted in the red boxes).

## Data Availability

Not applicable.

## Abbreviations

ART: Assisted Reproductive Technologies
BMI: Body Mass Index
C-section: Cesarean section
DOHaD: Developmental Origins of Health and Disease
GLM: Generalized Linear Models
HMO: Human Milk Oligosaccharides
IQR: Interquartile Range
NCDs: Non-communicable Diseases
OTUs: Operational Taxonomic Units
PCA: Principal Component Analysis
QC: Quality Control
WC: Waist Circumference
