# Supplementary material for: Prenatal and postnatal determinants in shaping offspring’s microbiome in the first 1000 days: study protocol and preliminary results at one month of life
Source: Ital J Pediatr. 2020 Apr 15;46:45. doi: 10.1186/s13052-020-0794-8 (PMC7158098; doi:10.1186/s13052-020-0794-8)
Supplement: Supplementary file 1 — Additional file 1: Table S1. Means of number of Reads Passing Filter quality, percentage of Reads classified to Genus level, Shannon diversity index, and identified species (OTUs) found: A) in the meconium (T0) and in fecal samples after 1 month (T1) of neonates; B) in the meconium of neonates born via cesarean section (CS) or vaginal delivery (VD); C) in the meconium of neonates born to normal weight (BMI < 25 Kg/m2) mothers affected by overweight or obesity (BMI ≥25 Kg/m2)), and D) in T1-samples of neonates fed with formula (FF) or breastfeeding (BF). [file 13052_2020_794_MOESM1_ESM.docx]

**Additional Table S1.**

A)

|  | Number Reads PF | % Reads Classified to Genus | Shannon Species Diversity | OTUs |
| --- | --- | --- | --- | --- |
| T0 | 77242.98 ^b^ | 88.07 ^a^ | 2.10 ^b^ | 354.98 ^a^ |
| T1 | 137393.65 ^a^ | 93.92 ^a^ | 2.30 ^a^ | 289.23 ^b^ |

B)

|  | Number Reads PF | % Reads Classified to Genus | Shannon Species Diversity | OTUs |
| --- | --- | --- | --- | --- |
| VD | 78463.48 ^a^ | 87.99 ^a^ | 2.07 ^a^ | 349.52 ^a^ |
| CS | 70191.22 ^a^ | 88.53 ^a^ | 2.25 ^a^ | 386.56 ^a^ |

C)

|  | Number Reads PF | % Reads Classified to Genus | Shannon Species Diversity | OTUs |
| --- | --- | --- | --- | --- |
| BMI<25 | 81008.07 ^a^ | 87.41 ^a^ | 2.10 ^a^ | 354.05 ^a^ |
| BMI>=25 | 66204.47 ^a^ | 90.67 ^a^ | 2.19 ^a^ | 382.07 ^a^ |

D)

|  | Number Reads PF | % Reads Classified to Genus | Shannon Species Diversity | OTUs |
| --- | --- | --- | --- | --- |
| BF | 131718.88 ^a^ | 93.94 ^a^ | 2.32 ^a^ | 286.79 ^a^ |
| FF | 140921.12 ^a^ | 93.65 ^a^ | 2.31 ^a^ | 294.88 ^a^ |

Values within a column with different superscript letters are significantly different (P<0.05; FDR<0.05).
