# Supplementary figures and images for: Prenatal and postnatal determinants in shaping offspring’s microbiome in the first 1000 days: study protocol and preliminary results at one month of life
Source: Ital J Pediatr. 2020 Apr 15;46:45. doi: 10.1186/s13052-020-0794-8 (PMC7158098; doi:10.1186/s13052-020-0794-8)

## Slide 1
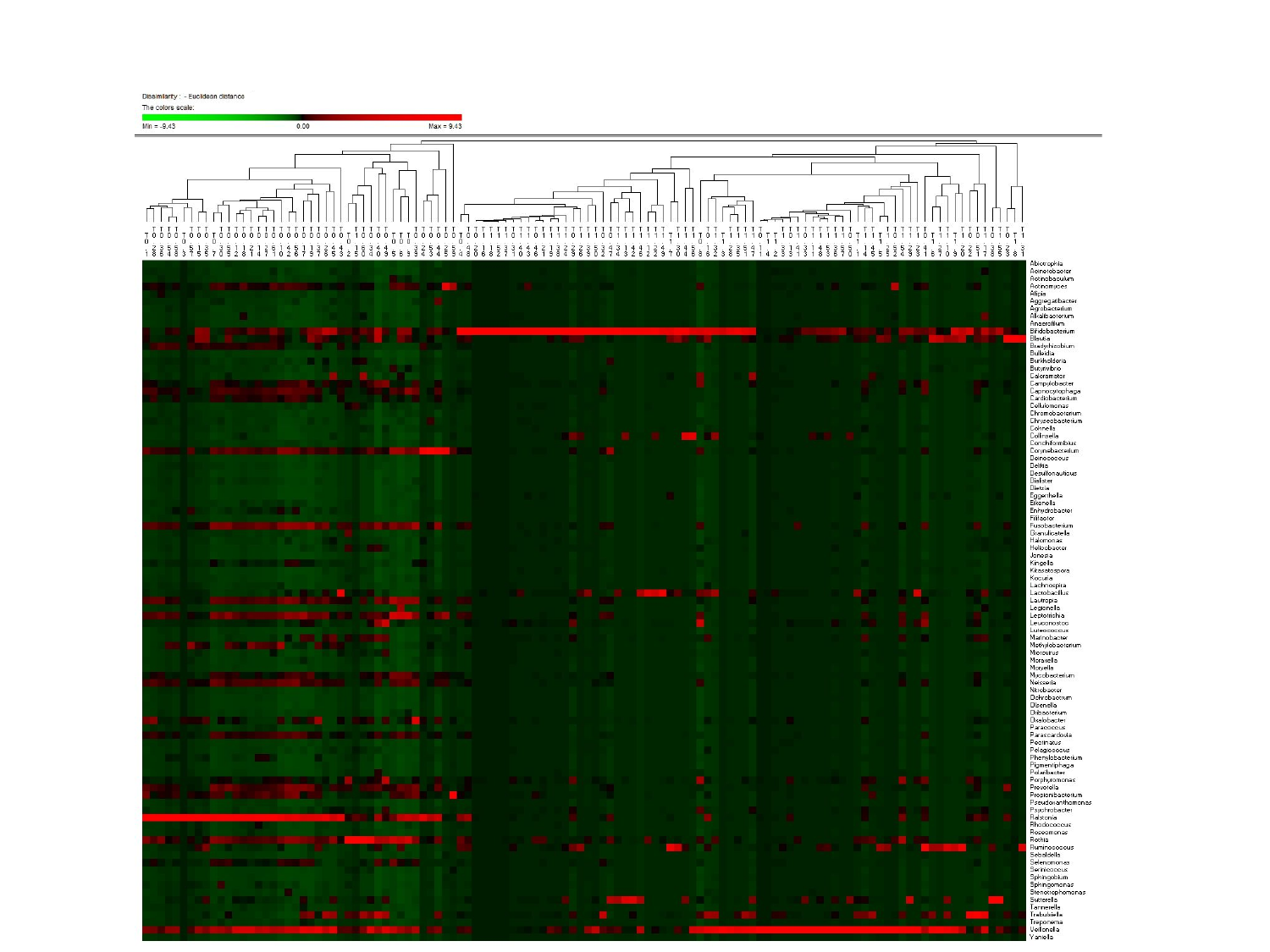

Supplement: Supplementary file 2 — Additional file 2: Figure S1a. Permutation analysis summarizing the genera with relative abundance > 0.1%, statistically different (p-value< 0.05; Student’s t-test), found in the meconium of neonates (T0) and after 1 month of age (T1). Figure S1b. Permutation analysis summarizing the genera with relative abundance > 0.1%, statistically different (p-value< 0.05), found in the meconium of neonates born to vaginal delivery (VD) or via cesarean section (CS; highlighted in the red boxes). Figure S1c. Permutation analysis summarizing the genera with relative abundance > 0.1%, statistically different (p-value< 0.05), found in the meconium of neonates born to normal weight (BMI < 25 Kg/m2) or mothers affected by overweight or obesity (BMI ≥25 Kg/m2; highlighted in the red boxes). Figure S1d. Permutation analysis summarizing the genera with relative abundance > 0.1%, statistically different (p-value< 0.05), found in fecal samples of neonates after 1 month (T1) fed with breast milk (BF) or formula (FF; highlighted in the red boxes). [file 13052_2020_794_MOESM2_ESM.zip › Figure S1a. Permut genera.pptx]

## Slide 1
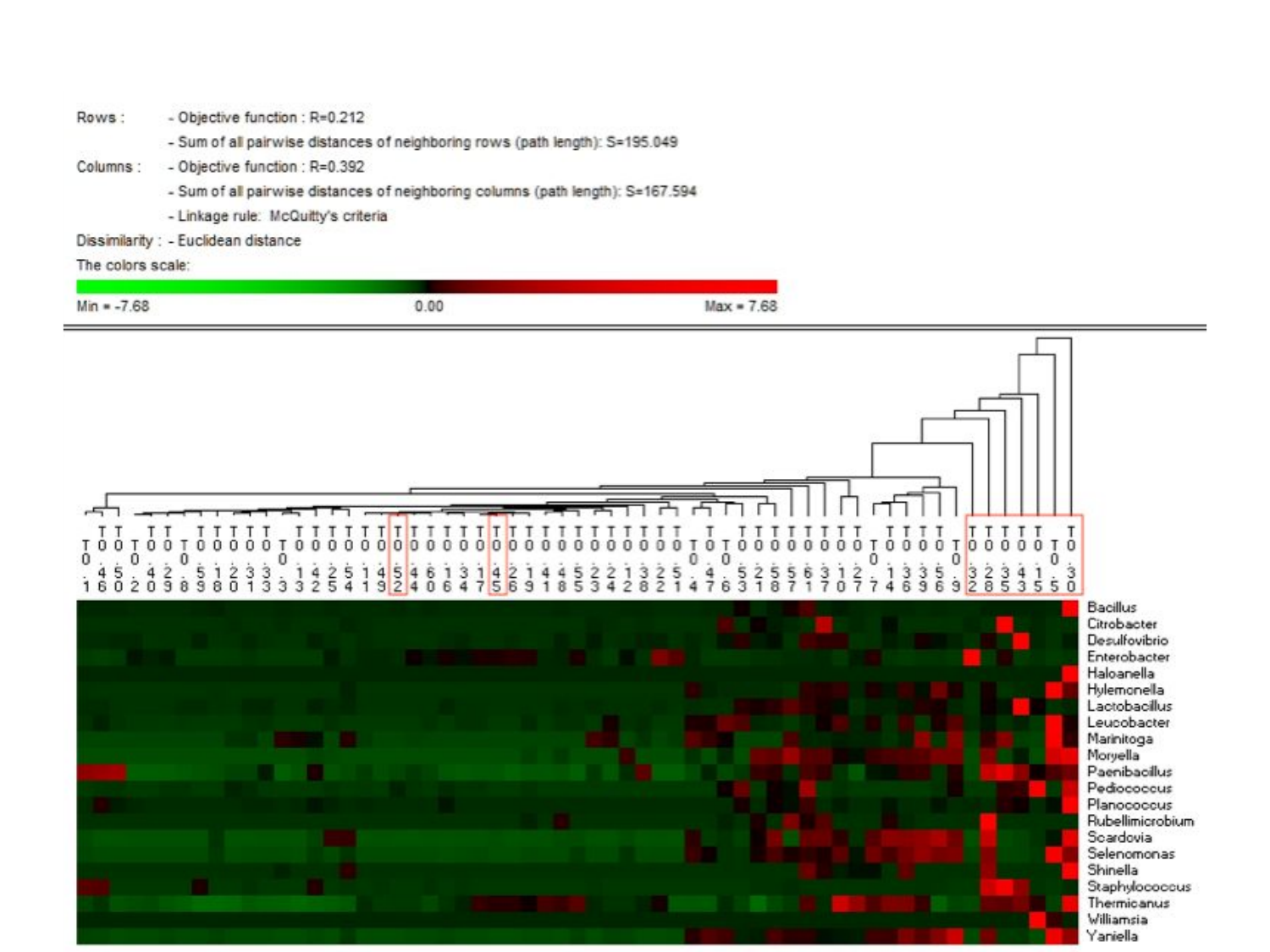

Supplement: Supplementary file 2 — Additional file 2: Figure S1a. Permutation analysis summarizing the genera with relative abundance > 0.1%, statistically different (p-value< 0.05; Student’s t-test), found in the meconium of neonates (T0) and after 1 month of age (T1). Figure S1b. Permutation analysis summarizing the genera with relative abundance > 0.1%, statistically different (p-value< 0.05), found in the meconium of neonates born to vaginal delivery (VD) or via cesarean section (CS; highlighted in the red boxes). Figure S1c. Permutation analysis summarizing the genera with relative abundance > 0.1%, statistically different (p-value< 0.05), found in the meconium of neonates born to normal weight (BMI < 25 Kg/m2) or mothers affected by overweight or obesity (BMI ≥25 Kg/m2; highlighted in the red boxes). Figure S1d. Permutation analysis summarizing the genera with relative abundance > 0.1%, statistically different (p-value< 0.05), found in fecal samples of neonates after 1 month (T1) fed with breast milk (BF) or formula (FF; highlighted in the red boxes). [file 13052_2020_794_MOESM2_ESM.zip › Figure S1b. Permut genera.pptx]

## Slide 1
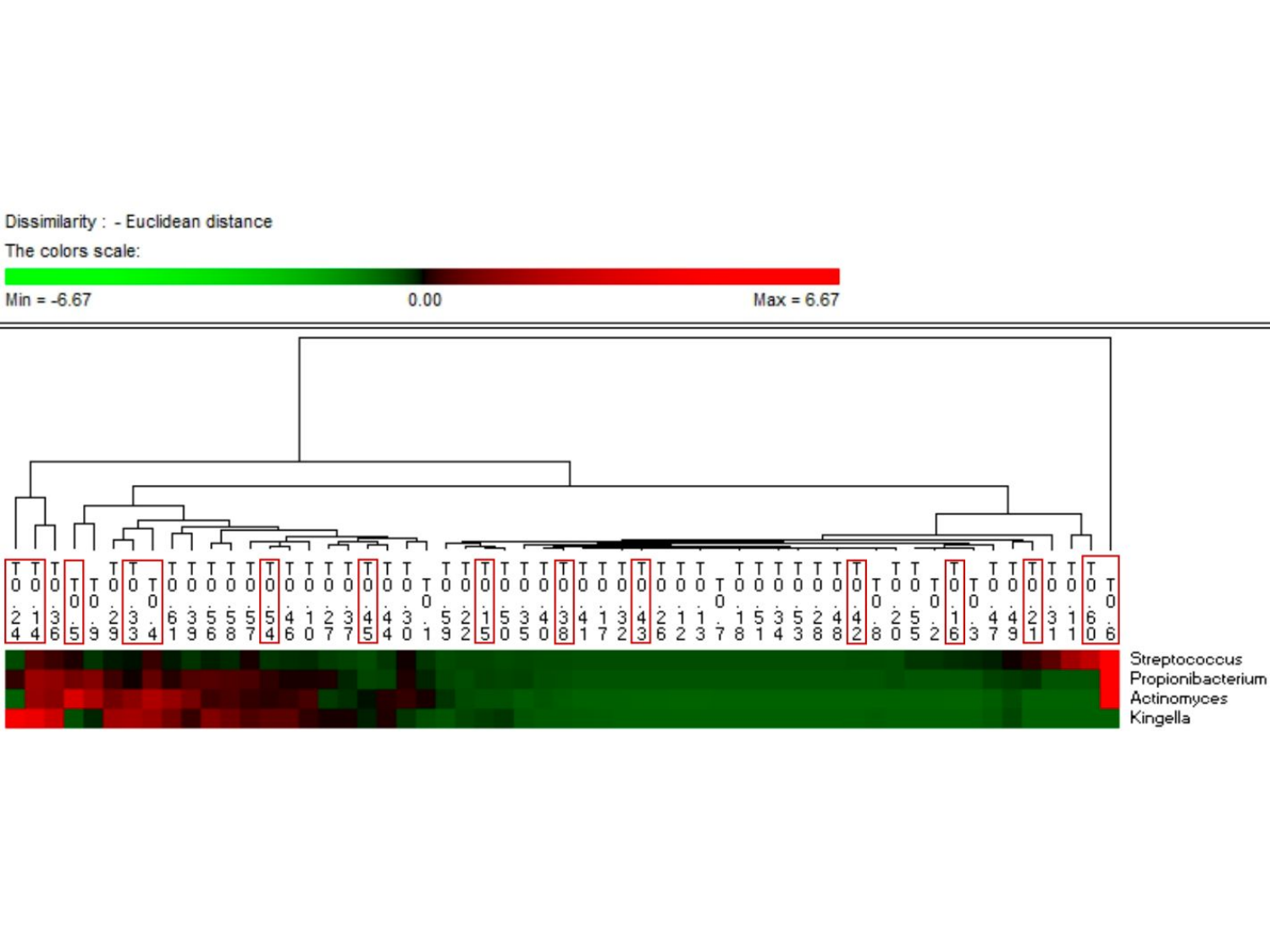

Supplement: Supplementary file 2 — Additional file 2: Figure S1a. Permutation analysis summarizing the genera with relative abundance > 0.1%, statistically different (p-value< 0.05; Student’s t-test), found in the meconium of neonates (T0) and after 1 month of age (T1). Figure S1b. Permutation analysis summarizing the genera with relative abundance > 0.1%, statistically different (p-value< 0.05), found in the meconium of neonates born to vaginal delivery (VD) or via cesarean section (CS; highlighted in the red boxes). Figure S1c. Permutation analysis summarizing the genera with relative abundance > 0.1%, statistically different (p-value< 0.05), found in the meconium of neonates born to normal weight (BMI < 25 Kg/m2) or mothers affected by overweight or obesity (BMI ≥25 Kg/m2; highlighted in the red boxes). Figure S1d. Permutation analysis summarizing the genera with relative abundance > 0.1%, statistically different (p-value< 0.05), found in fecal samples of neonates after 1 month (T1) fed with breast milk (BF) or formula (FF; highlighted in the red boxes). [file 13052_2020_794_MOESM2_ESM.zip › Figure S1c. Permut genera.pptx]

## Slide 1
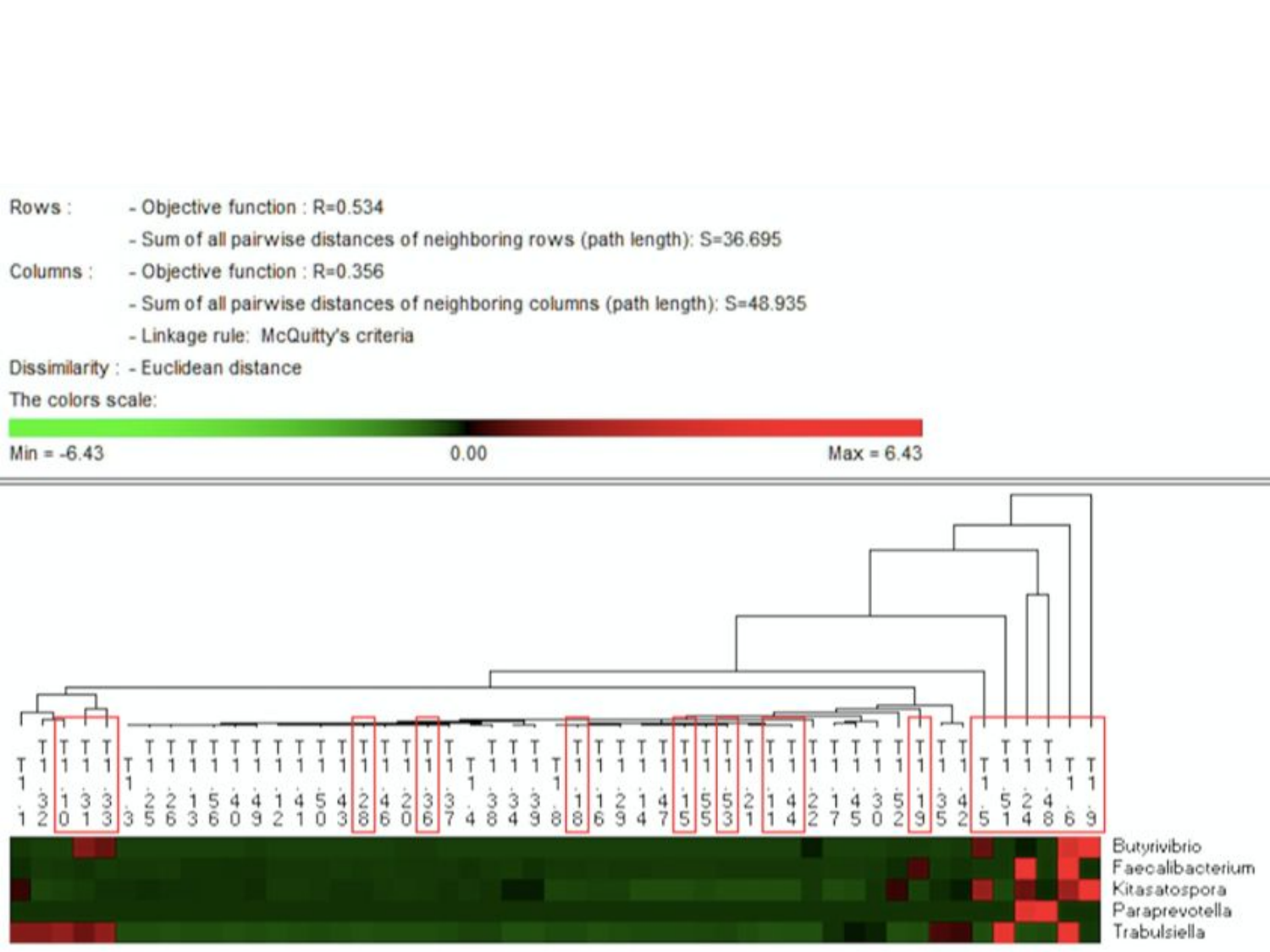

Supplement: Supplementary file 2 — Additional file 2: Figure S1a. Permutation analysis summarizing the genera with relative abundance > 0.1%, statistically different (p-value< 0.05; Student’s t-test), found in the meconium of neonates (T0) and after 1 month of age (T1). Figure S1b. Permutation analysis summarizing the genera with relative abundance > 0.1%, statistically different (p-value< 0.05), found in the meconium of neonates born to vaginal delivery (VD) or via cesarean section (CS; highlighted in the red boxes). Figure S1c. Permutation analysis summarizing the genera with relative abundance > 0.1%, statistically different (p-value< 0.05), found in the meconium of neonates born to normal weight (BMI < 25 Kg/m2) or mothers affected by overweight or obesity (BMI ≥25 Kg/m2; highlighted in the red boxes). Figure S1d. Permutation analysis summarizing the genera with relative abundance > 0.1%, statistically different (p-value< 0.05), found in fecal samples of neonates after 1 month (T1) fed with breast milk (BF) or formula (FF; highlighted in the red boxes). [file 13052_2020_794_MOESM2_ESM.zip › Figure S1d. Permut genera.pptx]

## Slide 1
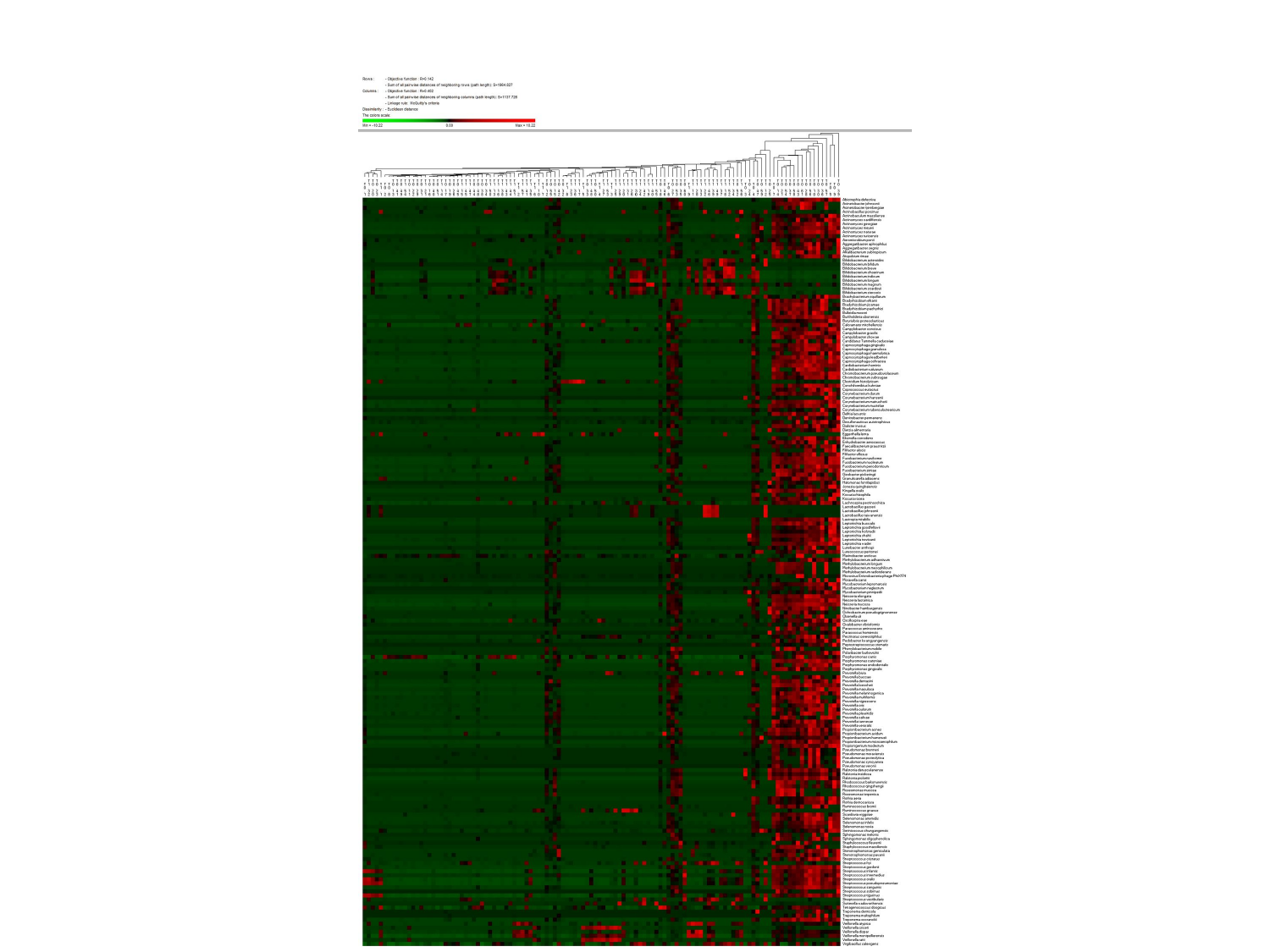

Supplement: Supplementary file 3 — Additional file 3: Figure S2a. Permutation analysis summarizing the species with relative abundance> 0.1%, statistically different (p-value< 0.05; Student’s t-test), found in the meconium of neonates (T0) and after 1 month of age (T1). Figure S2b. Permutation analysis summarizing the species with relative abundance> 0.1%, statistically different (p-value< 0.05; Student’s t-test), found in the meconium of neonates born to vaginal delivery (VD) or via cesarean section (CS; highlighted in the red boxes). Figure S2c. Permutation analysis summarizing the species with relative abundance> 0.1%, statistically different (p-value< 0.05; Student’s t-test), found in the meconium of neonates born to normal weight (BMI < 25) or overweight/obese mothers (BMI ≥25; highlighted in the red boxes). Figure S2d. Permutation analysis summarizing the species with relative abundance> 0.1%, statistically different (p-value< 0.05; Student’s t-test), found in fecal samples of neonates after 1 month (T1) fed with breast milk (BF) or formula (FF; highlighted in the red boxes). [file 13052_2020_794_MOESM3_ESM.zip › Figure S2a. Permut species.pptx]

## Slide 1
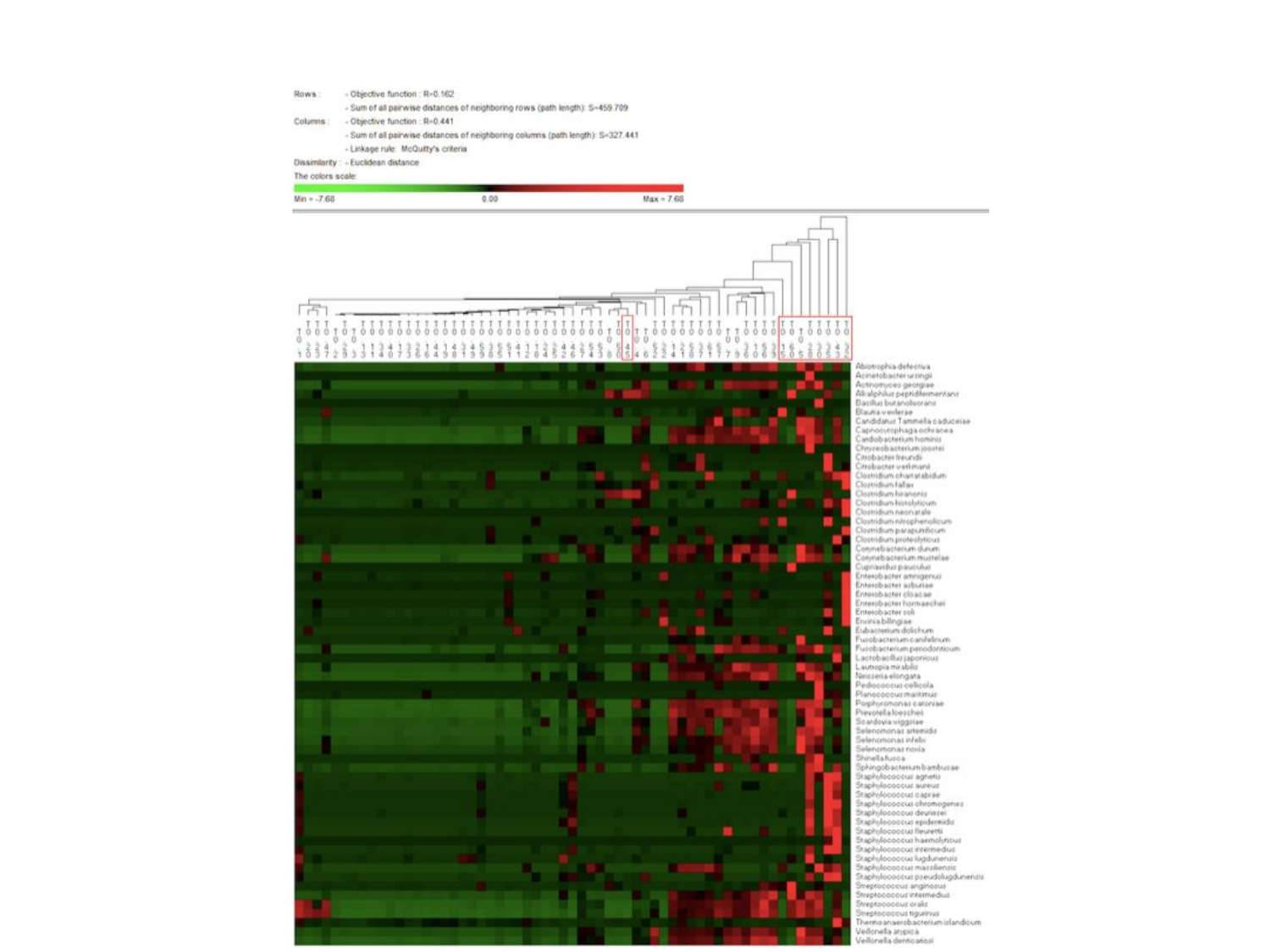

Supplement: Supplementary file 3 — Additional file 3: Figure S2a. Permutation analysis summarizing the species with relative abundance> 0.1%, statistically different (p-value< 0.05; Student’s t-test), found in the meconium of neonates (T0) and after 1 month of age (T1). Figure S2b. Permutation analysis summarizing the species with relative abundance> 0.1%, statistically different (p-value< 0.05; Student’s t-test), found in the meconium of neonates born to vaginal delivery (VD) or via cesarean section (CS; highlighted in the red boxes). Figure S2c. Permutation analysis summarizing the species with relative abundance> 0.1%, statistically different (p-value< 0.05; Student’s t-test), found in the meconium of neonates born to normal weight (BMI < 25) or overweight/obese mothers (BMI ≥25; highlighted in the red boxes). Figure S2d. Permutation analysis summarizing the species with relative abundance> 0.1%, statistically different (p-value< 0.05; Student’s t-test), found in fecal samples of neonates after 1 month (T1) fed with breast milk (BF) or formula (FF; highlighted in the red boxes). [file 13052_2020_794_MOESM3_ESM.zip › Figure S2b. Permut species.pptx]

## Slide 1
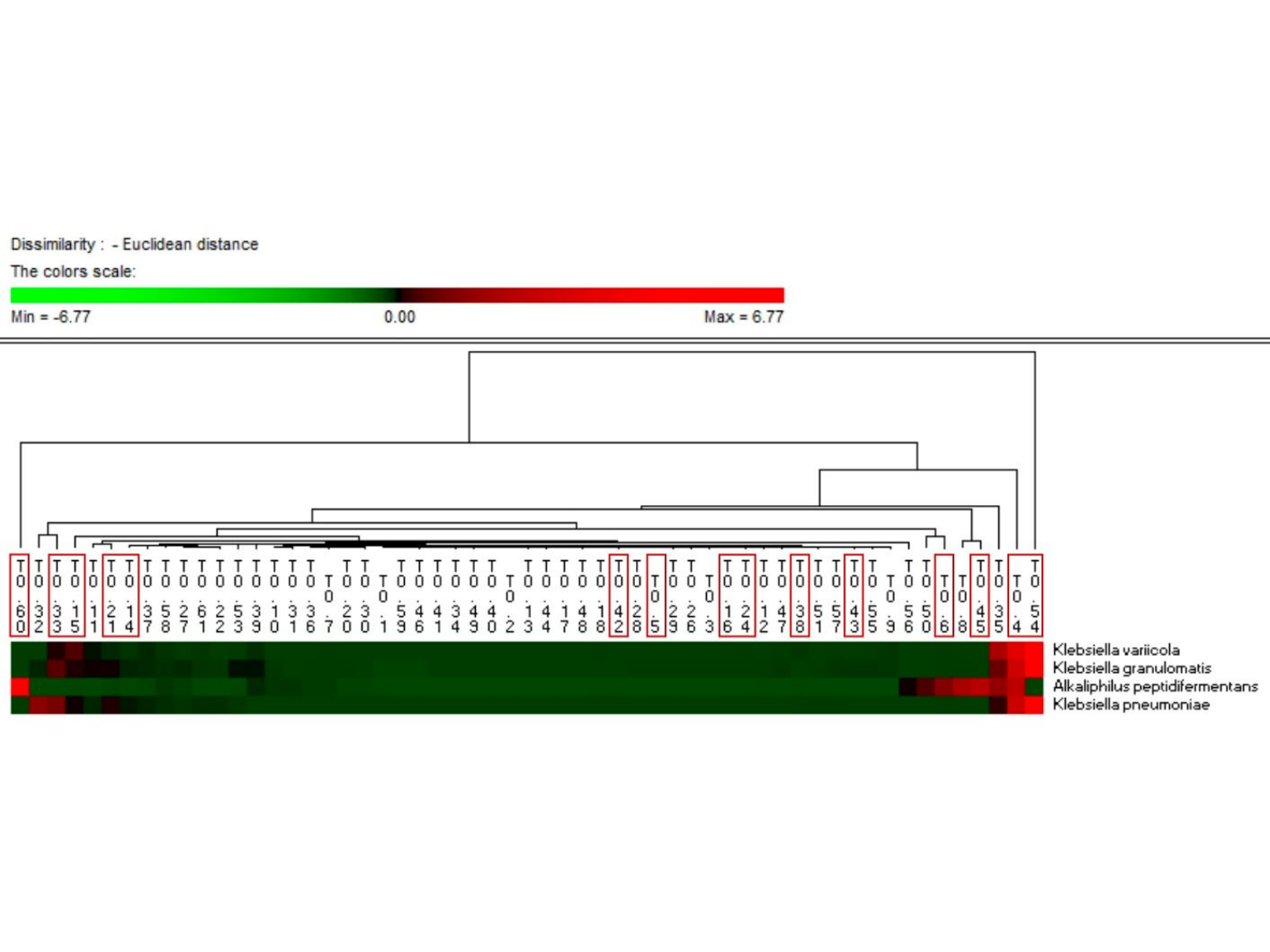

Supplement: Supplementary file 3 — Additional file 3: Figure S2a. Permutation analysis summarizing the species with relative abundance> 0.1%, statistically different (p-value< 0.05; Student’s t-test), found in the meconium of neonates (T0) and after 1 month of age (T1). Figure S2b. Permutation analysis summarizing the species with relative abundance> 0.1%, statistically different (p-value< 0.05; Student’s t-test), found in the meconium of neonates born to vaginal delivery (VD) or via cesarean section (CS; highlighted in the red boxes). Figure S2c. Permutation analysis summarizing the species with relative abundance> 0.1%, statistically different (p-value< 0.05; Student’s t-test), found in the meconium of neonates born to normal weight (BMI < 25) or overweight/obese mothers (BMI ≥25; highlighted in the red boxes). Figure S2d. Permutation analysis summarizing the species with relative abundance> 0.1%, statistically different (p-value< 0.05; Student’s t-test), found in fecal samples of neonates after 1 month (T1) fed with breast milk (BF) or formula (FF; highlighted in the red boxes). [file 13052_2020_794_MOESM3_ESM.zip › Figure S2c. Permut species.pptx]

## Slide 1
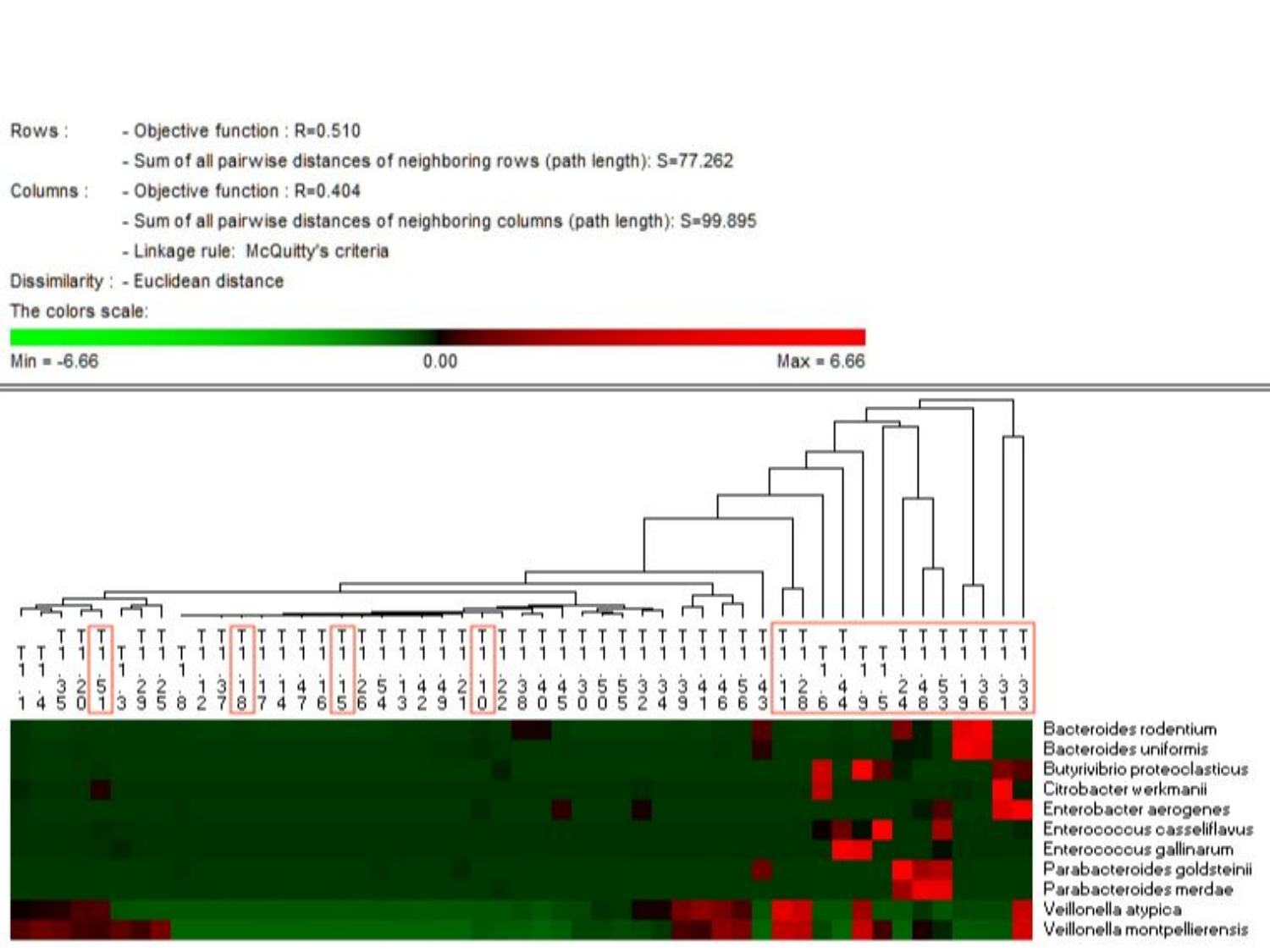

Supplement: Supplementary file 3 — Additional file 3: Figure S2a. Permutation analysis summarizing the species with relative abundance> 0.1%, statistically different (p-value< 0.05; Student’s t-test), found in the meconium of neonates (T0) and after 1 month of age (T1). Figure S2b. Permutation analysis summarizing the species with relative abundance> 0.1%, statistically different (p-value< 0.05; Student’s t-test), found in the meconium of neonates born to vaginal delivery (VD) or via cesarean section (CS; highlighted in the red boxes). Figure S2c. Permutation analysis summarizing the species with relative abundance> 0.1%, statistically different (p-value< 0.05; Student’s t-test), found in the meconium of neonates born to normal weight (BMI < 25) or overweight/obese mothers (BMI ≥25; highlighted in the red boxes). Figure S2d. Permutation analysis summarizing the species with relative abundance> 0.1%, statistically different (p-value< 0.05; Student’s t-test), found in fecal samples of neonates after 1 month (T1) fed with breast milk (BF) or formula (FF; highlighted in the red boxes). [file 13052_2020_794_MOESM3_ESM.zip › Figure S2d. Permut species.pptx]
